# An Efficient Electroporation Protocol Supporting In Vitro Studies of Oligodendrocyte Biology

**DOI:** 10.3390/mps8030064

**Published:** 2025-06-13

**Authors:** Yugo Ishino, Shoko Shimizu, Shingo Miyata

**Affiliations:** Division of Molecular Brain Science, Research Institute of Traditional Asian Medicine, Kindai University, Osaka-Sayama, Osaka 589-8511, Japan; shimizu@med.kindai.ac.jp (S.S.); smiyata@med.kindai.ac.jp (S.M.)

**Keywords:** OPC isolation, magnetic-activated cell sorting (MACS), OPC gene delivery, electroporation, dorsal root ganglion (DRG) explant, co-culture, myelination, neuron–glia interaction

## Abstract

Oligodendrocytes form myelin in the central nervous system, and their dysfunction can cause severe neurological symptoms, as large-scale analyses have highlighted numerous gene expression alterations in pathological conditions. Although in vivo functional gene analyses are preferable, they have several limitations, especially in large-scale studies. Therefore, standardized in vitro systems are needed to facilitate efficient and reliable functional analyses of genes identified in such studies. Here, we describe a practical and efficient method for oligodendrocyte precursor cell (OPC) isolation from mouse brains on postnatal day 6–8 and a gene delivery method for the isolated OPCs. By modifying the magnetic-activated cell sorting (MACS) procedure with reduced processing volumes, we simplified OPC isolation, allowing simultaneous handling of multiple samples and improving workflow efficiency. We also optimized electroporation parameters to achieve robust transfection efficiency with minimal cell death. Transfected OPCs are suitable for both monoculture-based differentiation assays and co-culture with dorsal root ganglion (DRG) explants, in which they reliably differentiate into mature oligodendrocytes and myelinate along the axons. This system enables stable and reproducible in vitro analysis of oligodendrocyte function, supports investigations into both intrinsic differentiation and neuron–glia interactions, and provides a powerful platform for oligodendrocyte research with efficient and timely gene manipulation.

## 1. Introduction

Oligodendrocytes (OLs) generate myelin sheaths around neuronal axons in the central nervous system (CNS) and enable fast and accurate signal propagation across neuronal networks. Moreover, evidence has demonstrated that OLs actively contribute to neuronal functions by supplying energy, releasing trophic factors, and regulating action potential conduction through dynamic myelin remodeling [1,2,3]. Recent studies have suggested that OL activity affects circuit synchronization [4,5], highlighting its broader role in shaping neuronal communication beyond structural support. Accordingly, OL dysfunction and altered gene expression have been increasingly reported in the pathogenesis of various neurodegenerative and neuropsychiatric disorders, including Alzheimer’s disease (AD) [6], Parkinson’s disease (PD) [7,8], and schizophrenia (SZ) [9], suggesting that OL malfunction plays a pivotal role in CNS pathology. While in vivo analyses provide critical insights into OL biology [10,11,12,13], they are unsuitable for rapid and systematic evaluation of gene expression data. In this context, efficient and reproducible in vitro systems remain indispensable as they offer a practical platform to gain initial functional insights into candidate genes for further in vivo validation. Thus, the importance of in vitro analyses highlights the need to standardize key procedures, such as cell isolation and gene delivery, which make in vitro OL analysis more accessible to a broader range of researchers.

For in vitro studies of OLs, oligodendrocyte precursor cells (OPCs) are commonly obtained from neonatal mouse brains or generated from pluripotent stem cells, such as iPSCs or NSCs [14,15,16]. However, these traditional isolation methods are technically demanding and often require highly specialized protocols or extended culture times, making them unsuitable for handling multiple samples in parallel. One of the remaining challenges for in vitro OPC studies is the lack of a reliable and easy method for introducing exogenous genes. Although electroporation-based and virus-based approaches have been employed previously, they generally require large input cell numbers or sophisticated handling skills [17,18,19,20]. Taken together, these challenges highlight the need to establish more accessible and efficient methods for investigating OL biology, especially in the context of gene function and disease modeling.

To address these issues, we established a scaled-down OPC isolation protocol using a magnetic-activated cell sorting (MACS) strategy targeting platelet-derived growth factor receptor alpha (PDGFRα), a well-characterized OPC-specific cell surface antigen [21,22]. This procedure reduces the buffer usage and simplifies the process, allowing the efficient processing of multiple samples simultaneously. Furthermore, we presented an optimized electroporation-based method that enables efficient gene delivery into primary OPCs, even at low cell densities, while preserving their viability and differentiation capacities. Moreover, this approach is compatible with previously reported neuron–OPC co-culture systems [23], enabling gene manipulation and functional studies of genes of interest involved in axon–glial interactions and myelination. We anticipate that this protocol will serve as a valuable tool for accelerating OL research, particularly in the context of genetically modified models and limited sample availability.

## 2. Experimental Design

**Figure 1:**
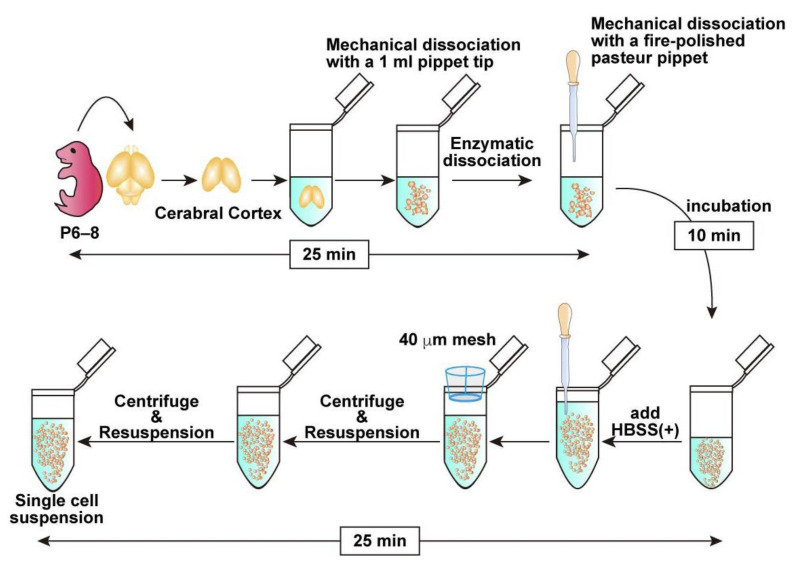
Schematic representation of the workflow from brain dissection to mechanical and enzymatic dissociation, followed by single-cell suspension preparation using a mesh filter with approximate times indicated at each major step, demonstrating that the entire procedure takes approximately 60 min.

**Figure 2:**
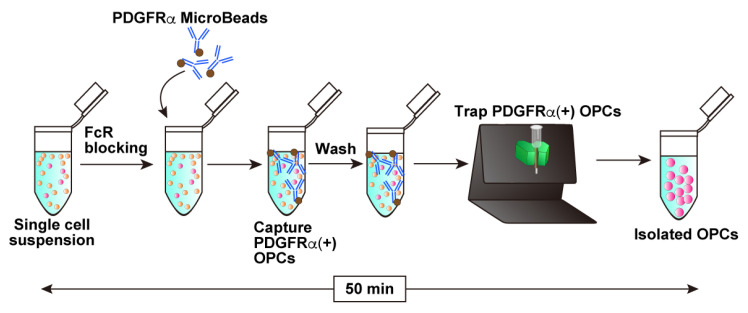
Schematic representation of PDGFRα-expressing OPC isolation following single-cell suspension preparation, illustrating the blocking step, antibody-based OPC capture, and elution of magnetically retained cells. The entire procedure takes approximately 50 min.

**Figure 3:**
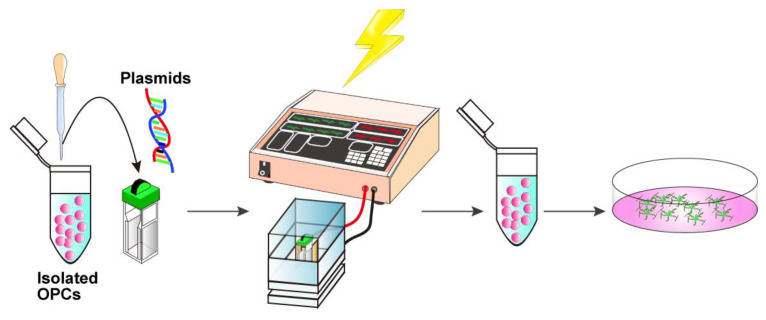
Schematic representation of the electroporation procedure. Isolated OPCs are gently mixed with 10 μg plasmid DNA, and the cell-DNA suspension is transferred into an electroporation cuvette. The suspension is then subjected to electrical pulses. Electroporated cells are immediately transferred to a culture medium.

**Figure 4:**
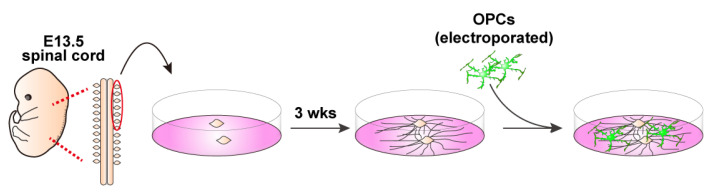
Schematic representation of embryonic DRG explant culture and co-culture with OPCs. DRGs are collected from E13.5 embryonic spinal cords and 3–4 explants are plated on PLO/MG-GFR-coated cover glasses. After 3 weeks of axonal outgrowth, OPCs are added for co-culture. Myelin formation is evaluated by immunostaining after 10 days of differentiation.

This protocol outlines a method for isolating OPCs from the brains of mice on postnatal days 6–8, followed by efficient gene transfection using an electroporation-based approach. In addition, we describe a co-culture system of OPCs with dorsal root ganglion (DRG) explants, enabling the investigation of neuron–glial interactions, particularly the formation of myelin sheaths along the DRG axons. The experimental flow and estimated time for each procedure are presented in schematic drawings: preparation of a single-cell mixture from postnatal brains (Figure 1), magnetic separation (Figure 2), electroporation steps (Figure 3), and DRG–OPC co-culture procedures (Figure 4).

### 2.1. Materials

- DMEM/F12 (ThermoFisher Scientific, Waltham, MA, USA, Cat. no.: 10565018)
- Neurobasal medium (ThermoFisher Scientific, MA, USA, Cat. no.: 12348017)
- Opti-MEM I Reduced Serum Medium (ThermoFisher Scientific, MA, USA, Cat. no.: 31985062)
- Hanks’ Balanced Salt Solution without Calcium and Magnesium (HBSS(−)) (ThermoFisher Scientific, MA, USA, Cat. no.: 14175053)
- HBSS with Calcium and Magnesium (HBSS(+)) (ThermoFisher Scientific, MA, USA, Cat. no.: 14025092)
- Poly-L-Ornithine (PLO) (Sigma, St. Louis, MO, USA, Cat. no.: A-004-M)
- Matrigel growth factor reduced (MG-GFR) (Corning, NY, USA, Cat. no.: 354230)
- Trace Elements B (1000×) (Corning, NY, USA, Cat. no.: 25-022-CI)
- B27 supplement (50×) (ThermoFisher Scientific, MA, USA, Cat. no.: 17504044)
- Platelet-derived growth factor (PDGF-AA) (Funakoshi, Tokyo, Japan, Cat. no.: 100-13A)
- Ciliary neurotrophic factor (CNTF) (Funakoshi, Tokyo, Japan, Cat. no.: 450-13)
- Neurotrophin-3 (NT3) (Funakoshi, Tokyo, Japan, Cat. no.: 450-03)
- Triiodothyronine (T3) (Sigma, MO, USA, Cat. no.: T6397)
- Nerve growth factor (NGF) (Merck, Darmstadt, Germany, Cat. no.: 01-125)
- 5-Fluoro-5′-deoxyuridine (FdU) (Sigma, MO, USA, Cat. no.: F8791)
- N-Acetyl-L-cysteine (NAC) (Sigma, MO, USA, Cat. no.: A9165)
- Isoflurane (FUJIFILM, Osaka, Japan, Cat. no.: 099-06571)
- 4% Paraformaldehyde Phosphate Buffer Solution (4% PFA) (FUJIFILM, Osaka, Japan, Cat. no.: 161-20141)
- Neural Tissue Dissociation Kit (P) (NTDK (P)) (Miltenyi Biotec, Bergisch Gladbach, Germany, Cat. no.: 130-092-628)
- CD140a (PDGFRα) MicroBead kit (Miltenyi Biotec, Bergisch Gladbach, Germany, Cat. no.: 130-101-502)
- Dead Cell Makeup Deep Red (DOJINDO, Kumamoto, Japan, Cat. no.: C556)
- AA3-PLP/DM20 (PLP) antibody [24]
- βIII-Tubulin antibody (Sigma, MO, USA, Cat. no.: T8578)
- GFP antibody (Abcam, Cambridge, UK, Cat. no.: ab13970)
- Olig2 antibody (Merck, Darmstadt, Germany, Cat. no.: MABN50)
- PiggyBac Transposon Vector (Funakoshi, Tokyo, Japan, Cat. no.: PB530A-2)
- Super PiggyBac Transposase Expression Vector (Funakoshi, Tokyo, Japan, Cat. no.: PB210PA-1)

### 2.2. Equipment

- MS columns (Miltenyi Biotec, Bergisch Gladbach, Germany, Cat. no.: 130-042-201)
- MiniMACS Separator (Miltenyi Biotec, Bergisch Gladbach, Germany, Cat. no.: 130-042-102)
- MACS MultiStand (Miltenyi Biotec, Bergisch Gladbach, Germany, Cat. no.: 130-042-303)
- Super Electroporator NEPA21 Type II (NEPA21) (NEPAGENE, Chiba, Japan)
- Nepa Electroporation Cuvettes 2 mm Gap (NEPAGENE, Chiba, Japan, Cat. no.: EC-002)
- Cuvette chamber (NEPAGENE, Chiba, Japan, Cat. no.: CU500)
- 13 mm cover glass (MATSUNAMI, Osaka, Japan, Cat. no.: C013001)
- 4 well multi-dish (ThermoFisher Scientific, MA, USA, Cat. no.: 176740)
- Pasteur pipette (AS ONE, Osaka, Japan, Cat. no.: 2-2045-01)
- Mini Cell Strainer II 40 μm (Funakoshi, Tokyo, Japan, Cat. no.: HT-AMS-04002)
- All-in-one fluorescence microscopes (KEYENCE, Osaka, Japan, Cat. no.: BZ-X810)

## 3. Procedure

### 3.1. Preparation of Cover Glasses and NTDK (P) Reagents

1. Place 13 mm cover glasses in each well of 4-wells chamber.
2. Coat with 350 μL of PLO at 37 °C for overnight.
3. Remove the PLO, rinse with distilled water twice, and dry the cover glasses.
4. Coat the cover glasses with Matrigel GFR (MG-GFR) and incubate in a CO_2_ incubator for ≥30 min.
5. Prepare Enzyme Mix 1 and Enzyme Mix 2 of NTDK (P) as described in the manual (Table 1).
6. Warm 500 μL Enzyme Mix 1 in a 1.5 mL tube at 37 °C, and keep Enzyme Mix 2 on ice until use.

### 3.2. Brain Collection and Cell Dissociation (See Figure 1; Estimated Time: 60 min)

1. Anesthetize a postnatal day 6–8 (P6–8) mouse with isoflurane and sterilize its body using 70% ethanol.
  **Note**: P6–8 mice are commonly used in this protocol because their tissue is easier to handle and typically yields sufficient numbers of OPCs. While younger mice can also be used, efficiency tends to decrease in older animals beyond this age.
2. Decapitate and collect the brain in cold HBSS (−).
3. Carefully dissect and collect the cortex. If possible, remove the meninges.
  **Note**: Meningeal removal is optional. Retained meninges do not affect the OPC purification efficiency. While removal of the meninges is preferable when feasible, prolonged efforts to remove them may reduce the overall cell yield. Therefore, if meningeal removal is perceived to be time-consuming, it is advisable to proceed promptly with tissue dissociation to preserve cell viability.
4. Transfer the collected brain tissue into 500 μL of pre-warmed Enzyme Mix 1 prepared in a 1.5 mL tube. Pipette up and down with a 1 mL pipette tip 7–10 times to mince the tissue into small pieces approximately 1 mm in size.
5. Incubate in the tube for 10 min at 37 °C.
  **Note**: Although rotation is recommended by the manufacturer’s protocol, it is not required under the present conditions.
6. Add 7.5 μL Enzyme Mix 2, tap the tube to briefly mix the solution, dissociate the tissue mechanically with a fire-polished Pasteur pipette and incubate at 37 °C for an additional 10 min.
  **Note**: Enzyme Mix 2 must be mixed before mechanical dissociation as the tissue solution tends to be very sticky because of the release of genomic DNA from dead cells. Complete dissociation is not required in this step. Remaining Enzyme Mix 2 can be stored at 4 °C and retains enzymatic activity for at least 1 month.
7. Add 750 μL HBSS (+). Then, further dissociate the tissue into smaller pieces by gently pipetting up and down into a single-cell suspension using a new fire-polished Pasteur pipette.
8. Pass half of the cell suspension through a 40 µm cell strainer into a new 1.5 mL tube to obtain a single-cell suspension. Subsequently, apply 700 μL of HBSS (+) to the strainer to collect any remaining cells on the mesh. Repeat the same procedure with the remaining half of the cell suspension, collecting in a separate 1.5 mL tube.
  **Note**: If approximately 1.0 × 10^5^ cells are sufficient for downstream applications, the second filtration step may be omitted.
9. Centrifuge the cell suspension at 400× *g* for 10 min at 4 °C and remove the supernatant carefully.
10. Resuspend the cell pellet in 1 mL of HBSS (+), then repeat the centrifugation under the same condition, and remove the supernatant carefully.

### 3.3. Magnetic Sorting with CD140a (PDGFRα) Microbead Kit (See Figure 2; Estimated Time: 50 min)

1. Resuspend the cell pellet in 80 μL of PBS containing 0.5% BSA (0.5% BSA/PBS) and add 7 μL of FcR Blocking Reagent supplied in the CD140a (PDGFRα) Microbead kit.
2. Incubate for 10 min at 4 °C.
3. Add 7 μL of CD140a (PDGFRα) MicroBeads and mix gently by pipetting.
4. Incubate for 10 min at 4 °C.
5. Wash the cell suspension by adding 1.2 mL of 0.5% BSA/PBS and centrifuge at 500× *g* for 10 min.
6. Remove the supernatant carefully and resuspend the cell pellet in 500 μL of 0.5% BSA/PBS.
7. Proceed to the following magnetic isolation procedure.
8. Place an MS column onto a MACS separator settled on a MACS Multistand.
  **Note:** MS columns are suitable for processing small volumes of cell suspensions. Prepare one MS column for one 1.5 mL cell suspension.
9. Apply 500 μL of 0.5% BSA/PBS to the column and allow the buffer to pass through completely.
10. Apply the whole cell suspensions to the column.
11. Wash the column with 500 μL of 0.5% BSA/PBS 3 times.
12. Remove the column from the MACS separator, apply 1 mL of Opti-MEM to the column, and collect the magnetically retained PDGFRα-positive cells by firmly plunging it into a 1.5 mL tube.
  **Note**: If the isolated cells are cultured directly without electroporation, use the culture medium for cell elution.
13. Count the collected cells. Typically, 1.0–2.0 × 10^5^ cells in total would be collected.
  **Note**: This cell number is from one MS column, meaning that 2.0–4.0 × 10^5^ cells would be collected from one mouse brain since cell suspensions were split into two 1.5 mL tubes. However, applying cell suspensions from the two split tubes to one MS column does not increase the total cell number for recovery.

### 3.4. In Vitro Electroporation with a NEPA21 Electroporator (See Figure 3; Estimated Time: 10 min)

1. Centrifuge the isolated cells at 500× *g* for 10 min at 4 °C. Discard the supernatant and gently resuspend the cell pellet in Opti-MEM at a density of 5 × 10^4^ cells per 90 μL.
  **Critical**: At this step, the Opti-MEM should be kept at room temperature because the chilled medium may cause condensation on the cuvette surface, which can reduce the electroporation efficiency.
2. Add 10 μg of DNA solution to 90 μL cell suspension, adjust the total volume to 100 μL with Opti-MEM, mix gently with a fire-polished Pasteur pipette, and transfer the entire cell–DNA mixture into an electroporation cuvette.
  **Critical**: Ensure that no bubbles are present in the cuvette. If bubbles are present, gently tap the cuvette until they disappear.
3. Firmly insert the cuvette into the cuvette chamber.
4. Perform electroporation using the NEPA21 system under the following conditions:
  Poring pulse, 150 V; pulse length, 1 ms; interval, 50 ms; number of pulses, 2; decay rate, 10%; pulse direction switch, none.
  Transfer pulse, 30 V; pulse length, 50 ms; interval, 50 ms; number of pulses, 5; decay rate, 50%; pulse direction switch, yes.
5. Immediately transfer the transfected cells into 500 μL of OPC proliferation medium (Table 2).
  **Optional**: Debris can be removed by passing the electroporated cell solution through a 40 μm mesh. However, a significant number of cells may be lost due to their retention on the mesh.
6. Plate the cells onto PLO/MG-GFR-coated 13 mm cover glasses placed in a 4-well multi-dish and culture in the OPC proliferation medium.
7. For OPC differentiation, replace the OPC proliferation medium with OPC differentiation medium (Table 2) on day 2. Replace half of the medium every other day until analysis.

### 3.5. DRG Explants and Transfected OPCs Co-Culture (See Figure 4)

1. Collect embryonic day 13 (E13.5) embryos from a time pregnant female mouse and collect DRGs from cervical and thoracic regions of the spinal cord.
2. Plate 3–4 explants onto a 13 mm cover glass coated with PLO/MG-GFR in DRG medium (Table 3). Replace half of the medium every other day. During the first week, add FdU at a final concentration of 10 μM to eliminate proliferating cells, such as glial cells.
3. Allow neuronal axons to extend radially. Axons are typically ready for co-culture with OPCs after 3 weeks of culture.
4. For the neuron–glia co-culture, isolate OPCs as described above, transfect them with EGFP-expressing plasmids using electroporation, and plate the cells onto the established DRG explant culture.
  **Note**: For long-term co-culture, OPCs were electroporated with 5 μg of a PiggyBac Transposon vector encoding EGFP and 5 μg of a Super PiggyBac Transposase expression vector.
  **Optional**: Debris can be removed by passing the electroporated cell solution through a 40 μm mesh. However, a significant number of cells may be lost due to their retention on the mesh.
5. Culture DRGs and OPCs in co-culture growth medium (Table 3) for 2 days.
6. On day 2, replace the medium with co-culture differentiation medium (Table 3). Continue replacing half of the medium every other day until analysis.

### 3.6. Immunocytochemical Staining and Dead Cell Labeling

#### 3.6.1. Dead Cell Labeling

This step may be omitted if cell viability assessment is not required for subsequent analyses.

1. Prepare 1000× Dead Cell Makeup stock solution according to the product manual.
2. Dilute the 1000× stock solution with the culture medium to prepare a 1× working solution.
3. Replace the culture medium with the 1× Dead Cell Makeup working solution and incubate in a CO_2_ incubator for 15 min at 37 °C.
4. Rinse the cells twice with PBS and proceed to the following immunostaining steps.

#### 3.6.2. Immunocytochemical Staining

1. Fix the cells with 4% PFA solution for 20 min at room temperature.
2. Wash the cells 3 times with PBS and permeabilize cells with PBS containing 0.5% Triton X-100 (PBT) for 10 min at room temperature.
3. Proceed to a blocking step by incubating PBT containing 10% normal donkey serum (blocking buffer) for 1 h.
4. Remove the blocking buffer and apply first antibodies against targets such as GFP, PLP, and βIII-tubulin. Incubate overnight at 4 °C.
5. Wash 3 times with PBS and apply fluorophore-conjugated secondary antibodies. Incubate the mixture for 2 h at room temperature.
6. Wash 3 times with PBS and mount the coverslips for microscopic observation.

## 4. Expected Results

### 4.1. Efficiency of OPC Isolation and Their Viability

**Figure 5:**
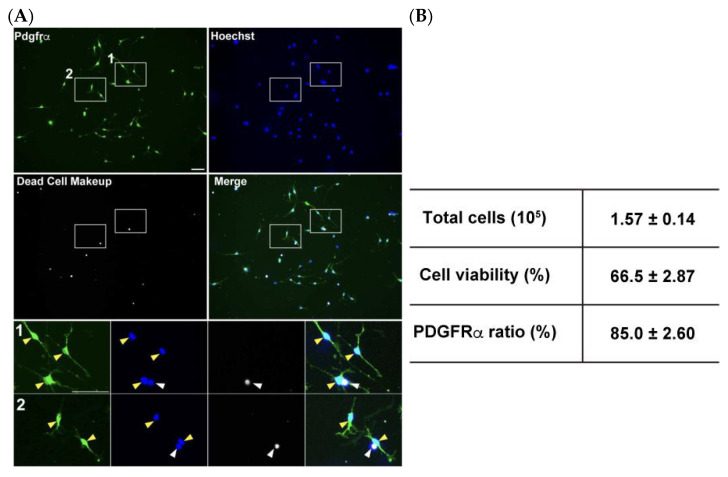
Efficient OPC isolation from the postnatal brain using a single MS column. (**A**) Collected OPCs were cultured for 2 days in the OPC proliferation medium and stained for PDGFRα (green), Dead cells (gray) and total cells with Hoechst (blue). Magnified images of boxed area are displayed in the small panels. White arrowheads indicate dead cells stained with Dead Cell Makeup stain, and yellow arrowheads indicate PDGFRα-positive cells. (**B**) Quantification data of total cells collected per MS column, cell viability, and the ratio of PDGFRα-positive cells among viable cells. Scale bars: 50 μm. Data are presented as the mean ± SD (*n* = 4).

The MACS cell isolation technique, which utilizes cell type specific antigens expressed on cell surfaces, has been widely used to isolate several neural cell types [25,26,27,28]. We modified the manufacturer’s protocol to adjust the buffer volume to be suitable for small tissue sizes derived from only one mouse brain, scaling down to approximately one-fourth of the recommended volume. The modified procedure typically yielded approximately 4 × 10^5^ total cells with two MS columns from a postnatal days 6–8 mouse brain (Figure 5A) within 2 h. These isolation efficiencies are comparable to those of a previous study in which an immunopanning strategy was adopted [15]. To confirm purification efficiencies for OPC isolation, collected cells were cultured in the OPC proliferation medium for 2 days, immunostained for PDGFRα, and combined with dead cell staining to assess cell viability. Despite the modest proportion of non-viable cells observed during processing and culturing, this protocol enabled highly efficient OPC enrichment, as 85 ± 4.5% of the surviving population demonstrates PDGFRα expression (Figure 5B). It is worth noting that PDGFRα expression may have partially decreased below the detection level during the 2-day culture. To further validate the enrichment of oligodendrocyte lineage cells, immunostaining for Olig2, a pan-oligodendrocyte marker, was performed. The results showed that nearly all viable cells (97.2 ± 0.98%) were Olig2-positive (Figure S1), confirming that MACS isolation using PDGFRα-conjugated beads is highly effective for obtaining an OPC-enriched population.

### 4.2. Gene Delivery into Isolated OPCs with an Electroporation Strategy

Primary cultured OPCs are one of the most challenging cell types for gene delivery. Owing to the significant advancements made in previous studies, virus-mediated gene delivery has become a reliable method, yielding favorable results in many cases [19,20]. However, this approach has certain limitations, including the time-consuming process of viral vector production and delayed gene expression following infection. Notably, some genes exert their functions exclusively during the early stages of OL differentiation [29]. In such cases, a system that enables rapid gene manipulation is essential to accurately assess gene function. For this purpose, we adopted an electroporation strategy using the NEPA21 electroporator, which allows researchers to manually adjust the electroporation parameters.

**Figure 6:**
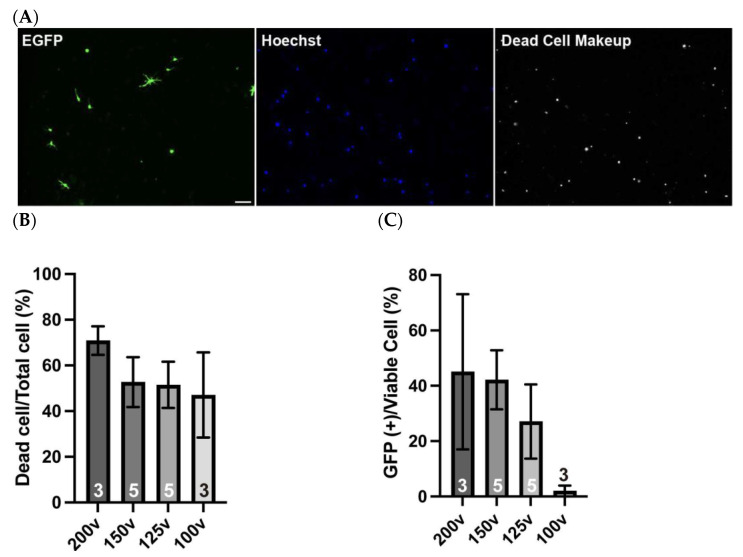

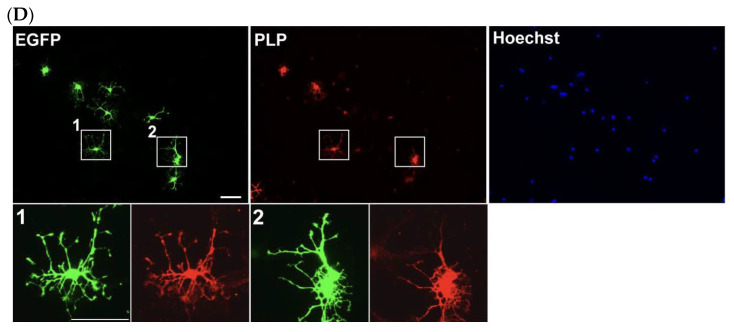
Electroporated cells retain their differentiation potential. (**A**) Transfected cells were cultured for 2 days and immunostained for EGFP (green) and dead cells (gray), with total nuclei labeled by Hoechst (blue). (**B**) Quantification of dead cells among all Hoechst-labeled cells. Voltage values indicate the poring pulse conditions. Higher voltages are associated with reduced cell viability. (**C**) Quantification of EGFP-positive cells among viable cells. Voltage values indicate the poring pulse conditions. (**D**) Differentiated OPCs are immunostained for EGFP (green) and PLP (red) after 4 days of differentiation. 36.5 ± 10.6% of EGFP-expressing cells differentiate into PLP-expressing mature oligodendrocytes. Magnified images of boxed area are displayed in the small panels. Scale bar: 50 µm. Data are presented as mean ± SD, and *n* values are shown in columns.

To achieve efficient DNA introduction into isolated OPCs, we systematically evaluated electroporation parameters using the NEPA21 apparatus. For optimization, we tested four distinct voltages, such as 200, 150, 125, and 100 V, as the poring pulse and found that 150V yielded a high transfection efficiency, with approximately 40% of viable cells expressing EGFP after two days in culture (Figure 6A). Both 150 V and 125 V conditions resulted in approximately 50% of total cells being viable after 2 days in culture (Figure 6B). As shown in Figure 5, isolated OPCs exhibited about 66% viability after 2 days without electroporation, indicating that 150 V and 125 V maintained roughly 75% viability relative to the non-electroporated controls. During the optimization steps, we found that although 200 V of the poring pulse yielded a transfection efficiency comparable to that of 150 V among viable cells, it was associated with a substantial number of dead cells across multiple experiments (Figure 6B). In some cases, more than 70% of the cells were dead in 2 days of culture and the reproducibility was poor, as evidenced by the large standard deviation observed across replicates (Figure 6C). These findings indicate that, despite the high efficiency in some trials, 200 V is not a reliable condition. In contrast, electroporation at 100 V resulted in minimal cell death. However, few or no EGFP-positive cells were observed (Figure 6C). Considering the balance between the transfection efficiency and cell viability, we concluded that 150 V was the optimal poring pulse condition for OPC electroporation.

For subsequent analysis, electroporated OPCs were cultured in the OPC differentiation medium for 4 days. OPC differentiation was assessed by immunostaining for PLP, a mature OL marker. The result demonstrated that a substantial number (36.5 ± 10.6%) of EGFP-positive cells were also positive for PLP, clearly indicating that electroporated cells retained their full capacity for OL differentiation (Figure 6D).

### 4.3. DRG Neuron–OPC Co-Culture for Neuron–Glia Interaction

Since OPCs differentiate into myelin-forming OLs, their capacity is typically assessed not only using monoculture differentiation assays, but also with co-culture systems with neurons [30,31,32]. To enable this evaluation, we employed a co-culture system using isolated and electroporated OPCs together with DRG neurons based on a previously established DRG explant culture model [23].

**Figure 7:**
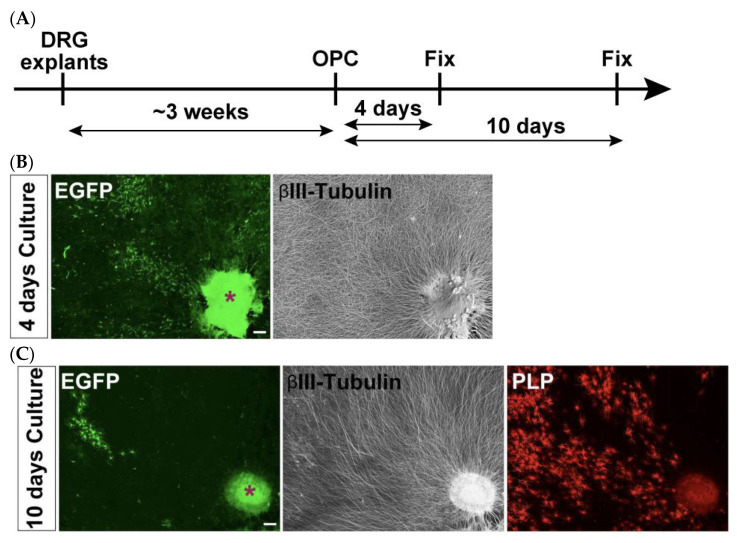
Transient EGFP expression supports short-term co-culture but limits long-term myelination assessment. (**A**) Schematic representation of the co-culture timeline. (**B**) Co-cultures were immunostained for EGFP (green) and βIII-tubulin (gray) after 4 days of differentiation. A substantial number of EGFP-positive cells are observed. (**C**) Co-cultures were immunostained for EGFP (green), βIII-tubulin (gray), and PLP (red) after 10 days of differentiation. Very few EGFP-positive cells remain. Note that, despite the loss of EGFP-positive cells, PLP-positive mature OLs are abundant. Asterisks indicate DRG explants. Scale bars: 200 µm.

Three to four DRG explants derived from E13.5 spinal cords were first cultured for 3 weeks to allow neurite extension. Subsequently, isolated and electroporated OPCs were plated onto the established DRG explants to initiate co-culture (Figure 7A). Immunostaining analyses were conducted 4 and 10 days after the induction of OPC differentiation to detect EGFP-expressing cells. Although many EGFP-positive cells were observed on day 4, their number was markedly reduced by day 10 (Figure 7B,C). Nevertheless, a substantial number of PLP-positive signals were still detected at this later time point, indicating the presence of differentiated OLs that were no longer expressing detectable levels of EGFP. This suggests that the initially observed EGFP fluorescence may have diminished over time, potentially due to reduced transgene expression or degradation. As such, the reduced number of EGFP-positive cells on day 10 did not allow for statistically meaningful quantifications.

**Figure 8:**
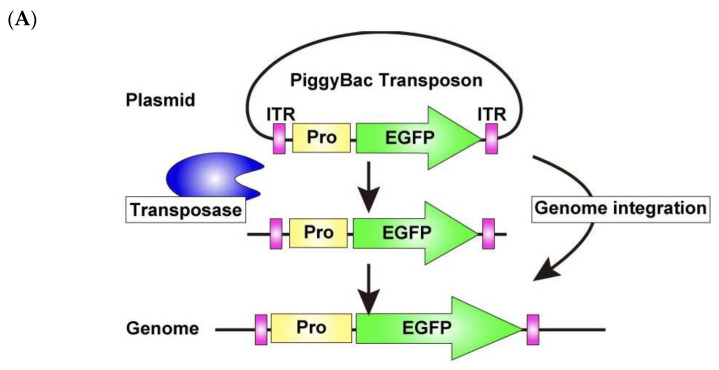

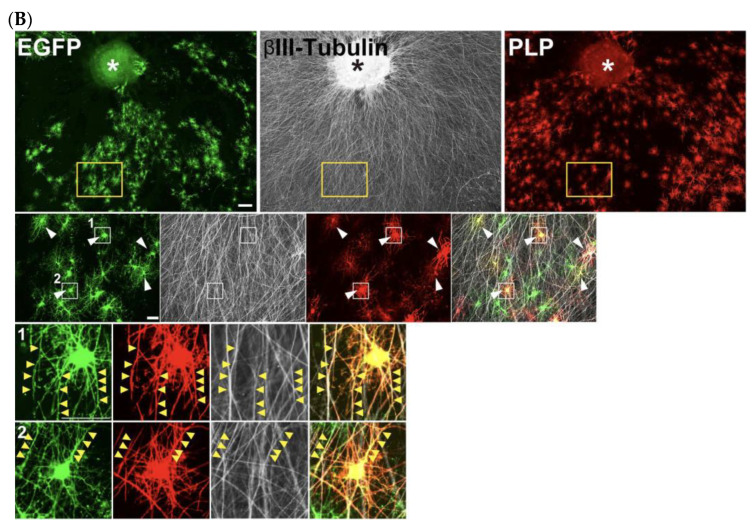
PiggyBac Transposon system enables long-term observation of exogenous gene expression in OLs within DRG–OPC co-cultures. (**A**) Schematic representation of the PiggyBac transposon system. DNA sequences flanked by two ITRs are excised from plasmids and integrated into the mouse genome using PiggyBac Transposase. (**B**) Co-cultures were immunostained for EGFP (green), βIII-tubulin (gray), and PLP (red) after 10 days of differentiation. Magnified images of the yellow boxed area are displayed in the small panel. A significant number of EGFP-expressing cells were also observed. Among them, 31.0 ± 2.73% of EGFP-positive cells are PLP positive, extending their processes and contacting with neuronal axons (white arrowheads). Furthermore, magnified images of the white boxed area are displayed in the smaller panels. Yellow arrowheads indicate EGFP-PLP double positive OL processes extending along DRG neuronal axons. Asterisks indicate the DRG explants. Scale bars: large panels, 200 μm; small panels, 50 μm. Abbreviations: ITR, inverted terminal repeat; Pro, promoter.

To overcome this limitation, we adopted a permanent labeling strategy, using the PiggyBac Transposon system [33,34] (Figure 8A), which enables efficient genomic integration of the transfected DNA and stable expression of the inserted genes. For electroporation, 5 μg each of the PiggyBac Transposon vector encoding EGFP and Super PiggyBac Transposase expression vectors was used. The OPCs were then cultured for 10 days under the differentiation condition. This approach led to a marked improvement, as a substantial number of EGFP-expressing cells remained even after 10 days of differentiation (Figure 8B). Furthermore, immunostaining for the OL differentiation marker PLP revealed that a substantial number (31.0 ± 2.73%) of EGFP-expressing cells are positive for PLP and exhibit close association with axons of DRG explants (Figure 8B), demonstrating that the PiggyBac Transposon system is a powerful tool for long-term gene manipulation, enabling efficient investigation of OL myelination in this co-culture.

## 5. Discussion and Conclusions

We described a protocol for the efficient isolation and genetic manipulation of oligodendrocyte precursor cells (OPCs), which overcomes several limitations of existing methods and provides a flexible platform for studying oligodendrocyte biology. This protocol is composed of three core components that collectively support a wide range of experimental applications.

First, we modified a MACS-based OPC isolation method that uses a reduced buffer volume, enabling the simultaneous processing of multiple samples derived from individual mouse brains. This approach allows researchers to isolate OPCs from single postnatal mice, facilitating parallel comparison across multiple experimental conditions. For instance, different pharmacological compounds can be administered to individual animals and analyzed simultaneously, for example, using omics approaches such as transcriptomics or proteomics. This feature is particularly advantageous in developmental studies, where it is often difficult to acquire a cohort of age-matched animals at the same time. Furthermore, the ability to process multiple samples in parallel makes it feasible to isolate and analyze OPCs from different genotypes of genetically modified mice under identical conditions, which is an important advantage for genotype-phenotype comparisons that were previously constrained by throughput limitations.

Second, we established an electroporation-based gene delivery protocol designed for freshly isolated OPCs. Previous protocols often relied on preset or undisclosed electrical parameters and required proprietary buffers [18], making it difficult to reproduce or customize electroporation conditions. In contrast, our method offers transparent and adjustable pulse settings using a commonly available system, allowing researchers to optimize electroporation conditions for their specific needs. Notably, our protocol includes newly established parameters that are effective even with limited cell numbers, which is particularly advantageous when working with primary cultures where large quantities of cells are difficult to obtain. This flexibility enhances reproducibility and accessibility across laboratories. Importantly, the detailed information provided here enables researchers to introduce gene constructs without the need for viral vectors, thereby reducing biosafety concerns and technical complexity.

Third, by integrating the DRG–OPC co-culture system with a PiggyBac transposon-based gene delivery system [33], we demonstrate that long-term gene expression can be maintained in differentiating oligodendrocytes. This feature is particularly beneficial for studying late-stage events such as myelin sheath formation or axon-glia interactions, which are often missed when transient gene expression systems are used. Furthermore, our system is expected to be compatible with inducible gene expression methods, such as the tetracycline-inducible (Tet-on/off) system [35]. This would enable researchers to activate gene expression at specific time points, such as during the late phase of differentiation or after myelination has occurred. Such temporal control is critical for dissecting gene functions involved in demyelination or axonal support. Moreover, because gene delivery occurs at the time of OPC isolation, gene expression is restricted to the OL lineage, without affecting neurons in the co-culture. This enhances the specificity and interpretability of downstream analyses.

Taken together, this protocol offers a robust and versatile platform for investigating gene functions in OL development and myelination. We believe this method will be a valuable resource for researchers seeking to dissect the molecular underpinnings of OL biology.

## 6. Troubleshooting

The most common pitfalls and their recommended solutions are summarized as follows:

- Low cell recovery after tissue dissociation:
  This often results from excessive pipetting force when trying to obtain a single-cell suspension. Other contributing factors include the generation of bubbles and the use of pipette tips with sharp edges. To address this, gentle pipetting is recommended, and fire-polished glass pipettes should be used to minimize shear stress and mechanical damage.
- Clogging of the MS column:
  Failure to properly resuspend the cell pellet after centrifugation can lead to column clogging. Ensure the suspension is homogeneous with no visible clumps before loading onto the MS column.
- Low transfection efficiency during electroporation:
  A common cause is the presence of air bubbles in the electroporation cuvette. Use a glass pipette to slowly and carefully apply the cell suspension to avoid introducing bubbles. If bubbles are present, they should be removed either by gently tapping the cuvette or by using a glass pipette to aspirate them.
- High cell mortality after electroporation:
  Cells should be promptly transferred to culture medium after electroporation to improve viability. Moreover, plasmid DNA should be purified using endotoxin-free kits to minimize toxicity.

## Figures and Tables

**Table 1:** Recipe for enzyme mix of NTDK (P).

Name	Recipe
Enzyme Mix 1	12.5 μL Enzyme P and 487.5 μL Buffer X
Enzyme Mix 2	20 μL Buffer Y and 10 μL Enzyme A

**Table 2:** Medium composition for OPC culture.

Name	Composition
OPC proliferation medium	DMEM/F12, B27 supplement, 10 ng/mL PDGF-AA, 10 ng/mL CNTF, 1 ng/mL NT3
OPC differentiation medium	DMEM/F12, B27 supplement, 10 ng/mL CNTF, 30 ng/mL T3, 5 μg/mL NAC

**Table 3:** Medium composition for DRG culture and co-culture.

Name	Composition
DRG medium	Neurobasal medium, B27supplement, 10 ng/mL NGF
Co-culture growth medium	Neurobasal medium:DMEM/F12 = 1:1 B27 supplement, N2 supplement, 10 ng/mL NGF, 10 ng/mL PDGF-AA, 10 ng/mL CNTF, 1 ng/mL NT3
Co-culture differentiation medium	Neurobasal medium:DMEM/F12 = 1:1 B27 supplement, N2 supplement, 10 ng/mL NGF, 10 ng/mL CNTF, 30 ng/mL T3, 5 μg/mL NAC

## Data Availability

The data that support the results are available from the corresponding author upon reasonable request.

## Supplementary Materials

The following supporting information can be downloaded at: https://www.mdpi.com/article/10.3390/mps8030064/s1, Figure S1. Most of the isolated cells are positive for Olig2.(A) OPCs collected by MACS were cultured for 2 days in the OPC proliferation medium and stained for Olig2 (green), Dead cells (gray), and total cells with Hoechst (blue). Magnified images of boxed area are displayed in the small panels. White arrowheads indicate dead cells stained with Dead Cell Makeup stain and yellow arrowheads indicate Olig2-positive cells. Quantification of Olig2-positive cells among viable cells is displayed within the Olig2 panel. Scale bars: 50 μm. Data are presented as the mean ± SD (*n* = 4).
