# Supplementary material for: An Efficient Electroporation Protocol Supporting In Vitro Studies of Oligodendrocyte Biology
Source: Methods Protoc. 2025 Jun 13;8(3):64. doi: 10.3390/mps8030064 (PMC12195875; doi:10.3390/mps8030064)
Supplement: Supplementary file 1 [file mps-08-00064-s001.zip › mps-3648672-supplementary.pdf]

(A)

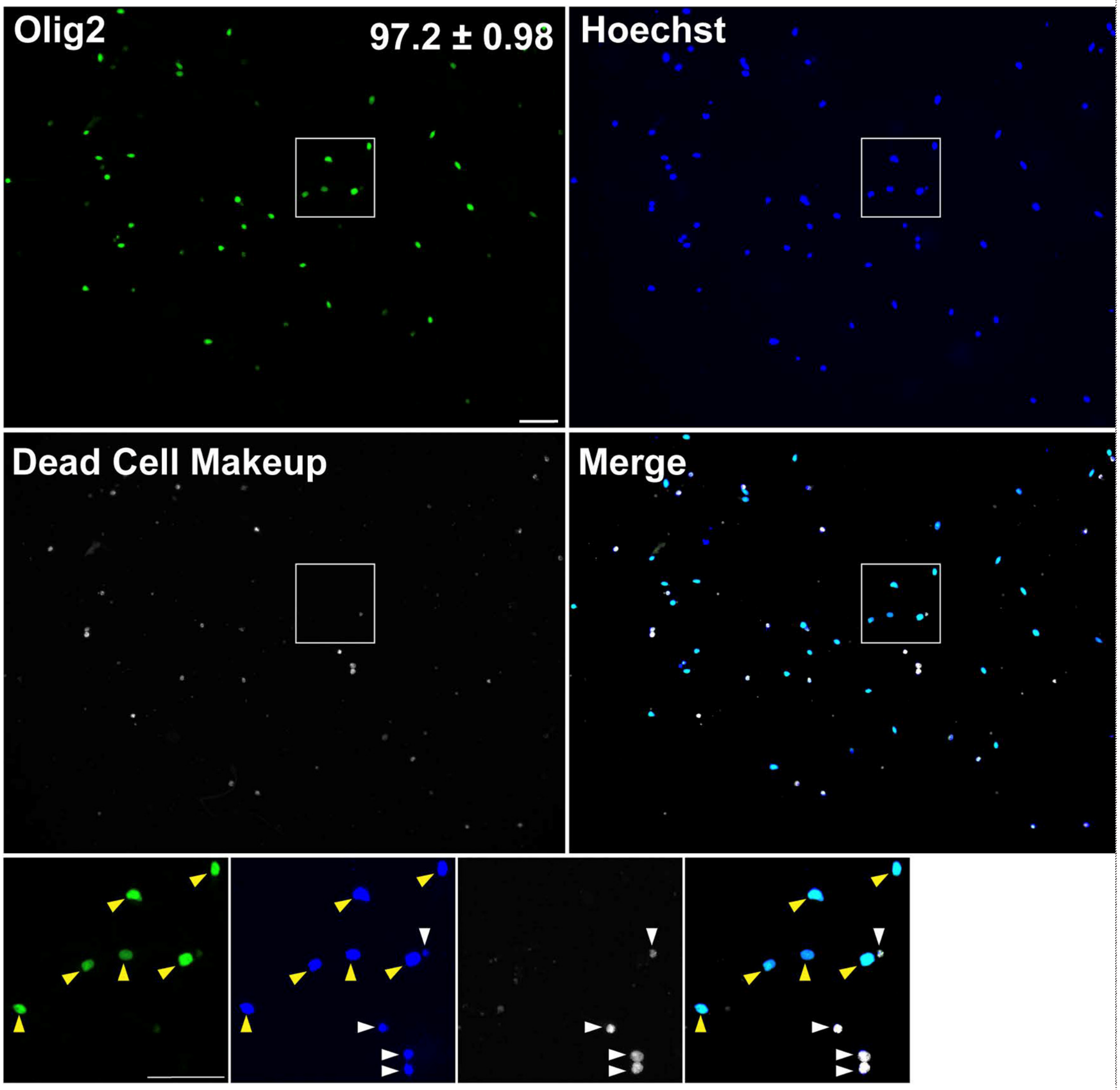

Figure S1. Most of the isolated cells are positive for Olig2. (A) OPCs collected by MACS were cultured for 2 days in the OPC proliferation medium and stained for Olig2 (green), Dead cells (gray), and total cells with Hoechst (blue). Magnified images of boxed area are displayed in the small panels. White arrowheads indicate dead cells stained with Dead Cell Makeup stain and yellow arrowheads indicate Olig2-positive cells. Quantification of Olig2-positive cells among viable cells is displayed within the Olig2 panel. Scale bars: 50 μm. Data are presented as the mean ± SD ($n = 4$).
